# Composition of White Rhinoceros Colostrum and Changes During Early Lactation to Mature Milk

**DOI:** 10.1002/zoo.21907

**Published:** 2025-06-06

**Authors:** Gernot Osthoff, Arnold Hugo, Petronel Nieuwoudt

**Affiliations:** ^1^ Department of Microbiology and Biochemistry University of the Free State Bloemfontein South Africa; ^2^ Department of Animal, Wildlife and Grassland Sciences University of the Free State Bloemfontein South Africa; ^3^ Care for Wild Rhino Sanctuary Nelspruit South Africa

**Keywords:** colostrum, desaturase, fatty acid, lactation, mineral, Perissodactyla

## Abstract

The proximate composition of milk from seven free‐ranging white rhinoceroses during the first 20 days of lactation is reported with detailed analysis of minerals and fatty acids. The composition of colostrum (day 1) is marked by a high content of dry matter at 23.8 g/100 g milk, mainly consisting of 18.0 g/100 g proteins, 5.6 g/100 g lactose, 0.6 g/100 g fat, 0.7 g/100 ash and 0.2 g/100 g NPN. The major minerals consisted of 24.0 mg/100 g Na, 55.2 mg/100 g K, 33.4 mg/100 g Ca, 10.3 mg/100 g P, 10.1 mg/100 g Mg and 0.4 mg/100 g Zn. The gross energy was 123.2 kCal/100 g. On day 2 the dry matter decreased to 11.9 g/100 g, specifically the ash and protein, and an increase of lactose, Ca and P. The change from colostrum to milk was complete at day two and transitional changes continued to day 5. Changes up to twenty days were minimal with lactose as main component at 6.6 g/100 g, followed by 1.3 g/100 g protein, 1.0 g/100 g fat, 0.2 g/100 g ash, 0.1 g/100 g NPN, 5.6 mg/100 g Na, 15.0 mg/100 g K, 53.3 mg/100 g Ca, 19.9 mg/100 g P and a gross energy of 41.4 kCal/100 g. At days 3 and 4 of lactation the fat content of the rhinoceros milk peaked at 1.6 g/100 g milk. The milk fatty acid composition was characterized by a high saturated content of 68–82%. Capric‐, lauric, myristic, and palmitic fatty acids were the major fatty acids, followed by oleic‐ and linoleic acids. Caprilic‐ and capric acid, as well as the sum of medium chain fatty acids content, steadily increased to stabilize after day 5. The Δ9‐desaturase 16 and ‐18 indexes reached peak activity at days 2–3 of lactation.

## Summary

- The colostrum phase of white rhinoceros milk ends after 2 days of lactation.
- The mature milk phase commences after 5 days.
- The fat content peaks at days 3–4.
- The Δ9‐desaturase 16 and ‐18 indexes peak at days 2–3.

## Graphical abstract

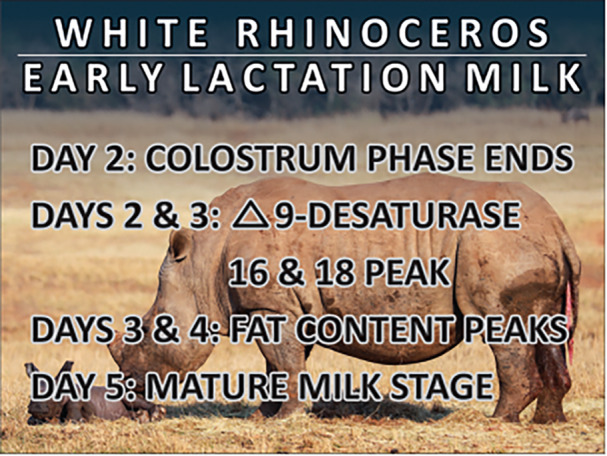

## 1 Introduction

The milk composition of white rhinoceros (*Ceratotherium simum*) was reported recently (Osthoff et al. 2021). It was observed that the milk composition did not undergo many significant changes over the study period of 0.5 to 18 months of lactation. The milk composition was also compared in depth with that of other perissodactyls such as the ass (*Equus asinus*) (Salimei et al. 2004; Salimei and Fantuz 2012), horse (*Equus caballos*) (Doreau and Martuzzi 2006; Markiewicz‐Keszycka et al. 2014), zebras (*Equus zebra* and *Equus burchellii*) (Oftedal and Jenness 1988) and tapir (*Tapirus terrestris*) (Van Nieuwenhove et al. 2014) and other Rhinocerotidae, the Indian rhinoceros (*Rhinoceros unicornis*) (Klös et al. 1972; Klös et al. 1974; Nath et al. 1993; Gimmel et al. 2018) and black rhinoceros (*Diceros bicornis*) (Gregory et al. 1965). It was concluded that differences in gross composition between these species were small, but that the milk of the Rhinocerotidae contain lower amounts of fat, higher lactose and equal protein, and therefore also the lowest gross energy, compared to the Equidae, while the Tapiridae milk contains the highest amounts of fat and protein, and lowest lactose.

There is evidence that dietary as well as phylogenetic factors amongst the Perissodactyla may contribute to differences in fatty acid composition of milk fat of the Equidae (Doreau and Martuzzi 2006; Markiewicz‐Keszycka et al. 2014) the Tapiridae (Van Nieuvenhove et al. 2014) and Rhincerotidae (Gimmel et al. 2018; Osthoff et al. 2008; 2022). They are known to contain a high content of medium chain fatty acids in the milk fat, often in excess of 30% of the total fatty acids (Osthoff et al. 2021; Osthoff 2022). In the Rhinocerotidae (Klös et al. 1972, 1974; Osthoff et al. 2022; Gimmel et al. 2018) the long chain fatty acids (C16 and C18) may be exceeded by the medium chain fatty acids. This is an indication that the S‐acyl fatty acid synthase thioesterase in the milk gland cells (Dils et al. 1977; Smith 1981) has a preference to terminate the elongation at 12 carbons or shorter. Apart from dietary effects, the milk fat composition of fatty acids may change during lactation, specifically in the evolution from colostrum to transitional milk as was observed in the milk of the cow (Wilms et al. 2022; O'Callaghan et al. 2020), ewe (Povlikova et al. 2010), sow (Csapo et al. 1996; Ren et al. 2022) and human (Kovács et al. 2005; Kuipers et al. 2012).

Apart from unsaturated fatty acids in the milk fat having a dietary origin, they are also produced by the action of Δ9‐desaturases that desaturate fatty acids at carbon 9 (Griinari et al. 2000; Corl et al. 2001). It is also important in the synthesis of conjugated linoleic acid. A progressive increase in the activity of this enzyme was shown in cow's milk over the length of lactation (Soyeurt et al. 2008) and it might also be affected by the season (Lock and Garnsworthy 2003). Differences during the first days of lactation have not been reported.

In almost all mammals, colostrum contains a high content of salts and proteins which pass into the lumen of the mammary alveolus through the junctions of the epithelial cells. Once the synthesis and secretion of the milk nutrients is activated, the junctions are closed and the synthesized nutrients are secreted through the cellular route (Nguyen & Neville 1999). This process takes at least 24–48 h (Allen et al. 1991) but may last three to 5 days (Scano et al. 2014). Most of the information on milk composition of the Perissodactyl deals with mature milk and little is known about the colostrum. Reports on milk from horse (Solaroli et al. 1993), donkey (Yang et al. 2024) and white rhinoceros (Osthoff and Nieuwoudt 2024) limit the change‐over time to 2–3 days.

We report the changes of milk composition during the first 8 days of lactation in the white rhinoceros. Apart from changes in the macro‐components, changes of micro‐components, such as fatty acids, indicate the involvement of specific metabolic reactions. Some of the data is observed for the first time in a member of the Perissodactyla.

## 2 Materials and Methods

The research reported here complies to the guidelines of the American Society of Mammalogy (Sikes 2016) and the Animals Research Ethics of the University of the Free State (UFS‐AED2019/0052), Permission to do research in terms of section 20 of the Animal diseases act, 1984 (Act no. 35 of 1984) of South Africa (permit reference 12/11/1/4), Threatened or Protected Species Regulations (TOPS) (standing permit no. S07969, and registration certificate no. 29414) and South African National Parks Material Transfer Agreement 003/24.

Milk was obtained from seven white rhinoceroses of the Care for Wild Rhino Sanctuary, Nelspruit, Mpumalanga province of South Africa. The animals were in good health and roamed on Legogote Sour Bushveld (Mucina and Rutherford 2006), which was supplemented with lucern and teff (ratio 1:2) throughout the year. It was the first parturition for each of the animals, Timbi (age 10 years, parturition December 2022), Olive (9 years, parturition August 2022), Tana (9 years, parturition November 2023), Wyntir (8 years, parturition February 2022), River (8 years, parturition March 2022), Twinkle (7 years, parturition November 2022) and Sibeva (7 years, parturition May 2023). The rhinoceroses were tame so that tranquilization and a milk letting agent was not required. Milk was drawn by palpation of the teats while sustained pressure was exerted on the udder. Teats were milked out, producing 30–50 mL milk per teat and milk from each teat was collected separately. Milk was frozen immediately at ‐20°C and kept frozen until analysed. Milk was thawed and mixed by swirling in a water bath set to 39°C.

Approximately 1.0 g white rhinoceros milk was used to determine the water content gravimetrically after drying for 2–3 h at 105°C in a forced convection drying oven (AOAC (1990). After incineration for 2 h at 550°C, the ash was dissolved in 30% nitric acid, and the minerals were analyzed by inductively coupled plasma optical emission spectroscopy (ICP‐OES) by the Center of Groundwater Studies, University of the Free State (American Public Health Association 2005).

Non protein nitrogen (NPN) was obtained by selective precipitation (Igarashi 1995) and the nitrogen content of NPN and crude protein (CP) determined with a Leco® nitrogen (N) analyser (LECO CORPORATION 2001). The protein content was calculated by subtracting NPN from the total nitrogen and multiplying the nitrogen (N) content with a factor of 6.38.

Fat was extracted quantitatively according to Folch et al. (1957) with chloroform and methanol in a ratio of 2:1 (v/v). Total extractable fat content was determined gravimetrically and expressed as g fat/100 g milk. Fatty acids were extracted from the total fat and fatty acid methyl esters (FAME) were prepared by transesterification with 0.5 N NaOH in methanol and 14% boron trifluoride in methanol (Park and Goins 1994). The FAME were quantified by gas chromatography with a flame ionization detector and a fused silica capillary column, Chrompack CPSIL 88 (100 m length, 0.25 mm ID, 0.2 μm film thickness) in a using a Varian 430 chromatograph. The column temperature was 40°C–230°C (hold 2 min; 4°C/minute; hold 10 min) and total run time was 59.5 min. Injection of the solution of FAME in hexane (1 μl) was by a Varian 8400 Autosampler (Varian Inc. Walnut Creek, CA, USA) with a split ratio of 100:1. The injection port and detector were maintained at 250°C and hydrogen at 45 psi was the carrier gas with nitrogen as makeup gas. Recorded was with Galaxy Varian Star Chromatography Software (Version 6.41). Since nonadecanoic acid (C19:0) was not detected in the samples under study it was used as internal standard. Identification of sample FAME was according to the relative retention times of FAME standards obtained from Supelco (Supelco 37 Component FAME Mix 47885‐U together with C18:1c7, C18:2c9t11, C19:0, C22:5). Desaturation indices were calculated as the ratio of monounsaturated fatty acid to the corresponding saturated fatty acid.

Saccharides were extracted from milk with 500 µL 25% trichloroacetic acid per 1 mL milk sample and filtered through Nanosep 3 K MF Centrifugal Devices (Pall Life Sciences, Michigan, USA) in an Eppendorf centrifuge at 13000 rpm. De‐fatted and de‐proteinized extracts were prepared by centrifugation at 3000 × g in Ultrafree‐CL (UFC4 LCC 25) filter devices (Millipore, Johannesburg, South Africa). The filtrate (10 μL) was analysed with a Waters Breeze High Performance Liquid Chromatography system fitted with Biorad Aminex 42 C (300 × 7.8) mm (Pall Life Sciences, Ann Arbor MI, USA) and Waters Sugar Pak 1 (300 × 7.8) mm (Microsep, Johannesburg, South Africa) columns. Chromatography was performed at 84°C and detection of analytes was with a differential refractive detector. De‐ionized water was the mobile phase at a flow rate of 0.6 mL/min. Quantification was according to lactose, glucose and galactose as standards.

The milk energy content was calculated according to Perrin (1958) and using the contents of milk fat, carbohydrates and crude protein obtained with the methods described above. The formula to calculate the GE was:

GE=(9.11kcal/gx%Fat+5.86kcal/gx%Protein+3.95kcal/gx%Carbohydrate).

Individual scatterplots of time into lactation against individual chemical attributes were constructed. In an attempt to determine the relationship between time into lactation and chemical attributes (NCCS; Addinsoft 2020), exponential, linear, logarithmic and polynomial regressions were alternately fitted to each graph to determine the best fit (with respect to the R^2^ value) between time into lactation and chemical attribute. Principal components analysis (PCA) was carried out on Metaboanalyst (version 6.0; Pang et al. 2024).

## 3 Results and Discussion

**Figure 1:**
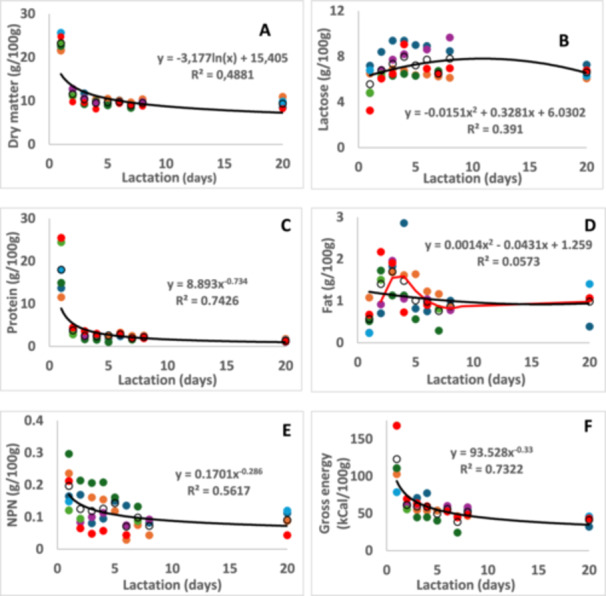
The changes of dry matter (A), lactose (B), protein (C), fat (D), NPN (E) and gross energy (F) in white rhinoceros milk during early lactation with best‐fit trend lines and moving average line (red) for the fat content. The interindividual variation of changes over lactation is shown for each animal: Average (O), Olive (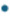), Twinkle (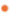), Timbi (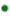), Sibev a(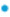), Tana (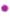), River (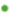), Wyntir (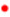).

**Figure 2:**
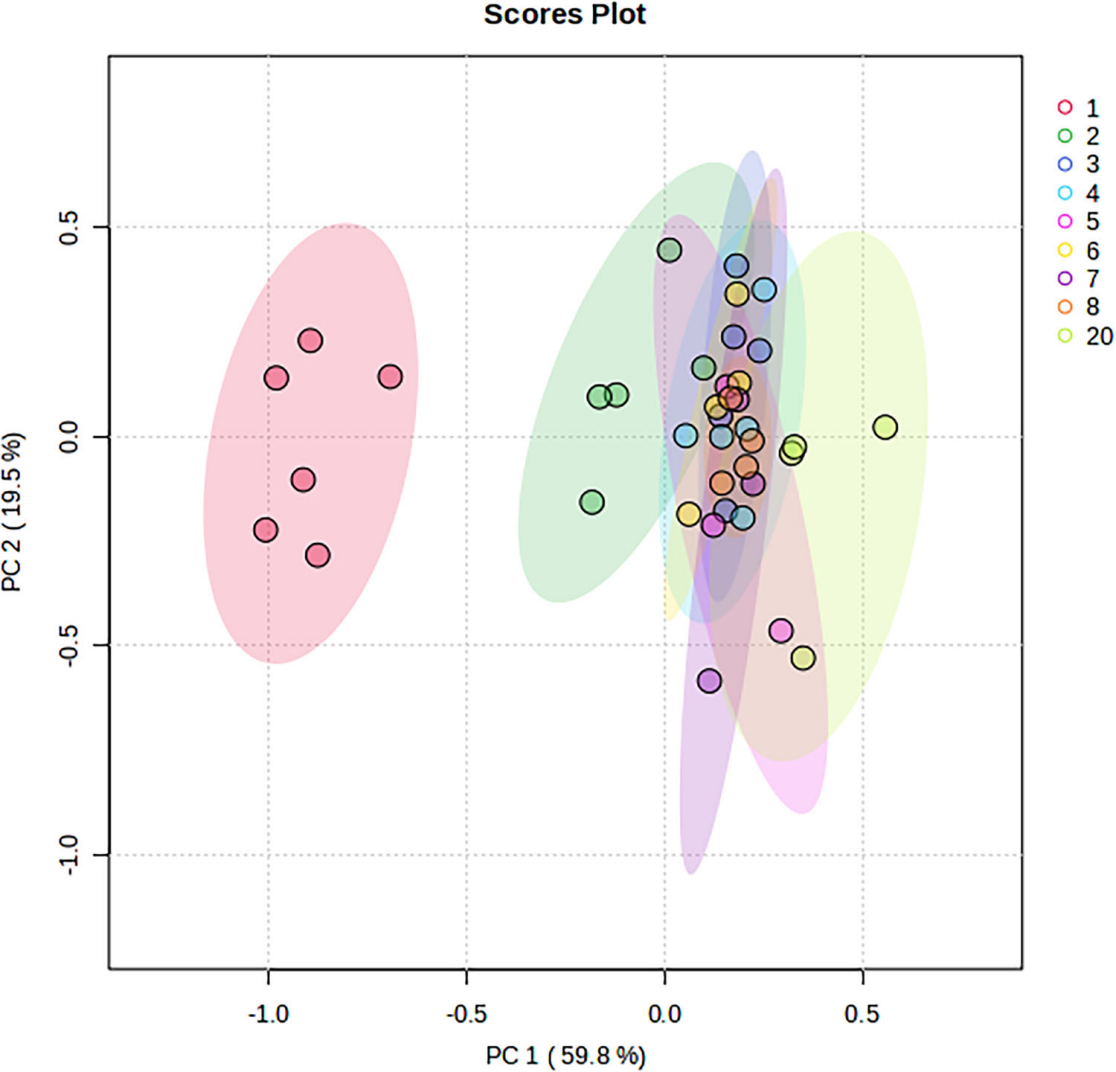
Principal components analysis of all the proximate components per day of lactation.

**Figure 3:**
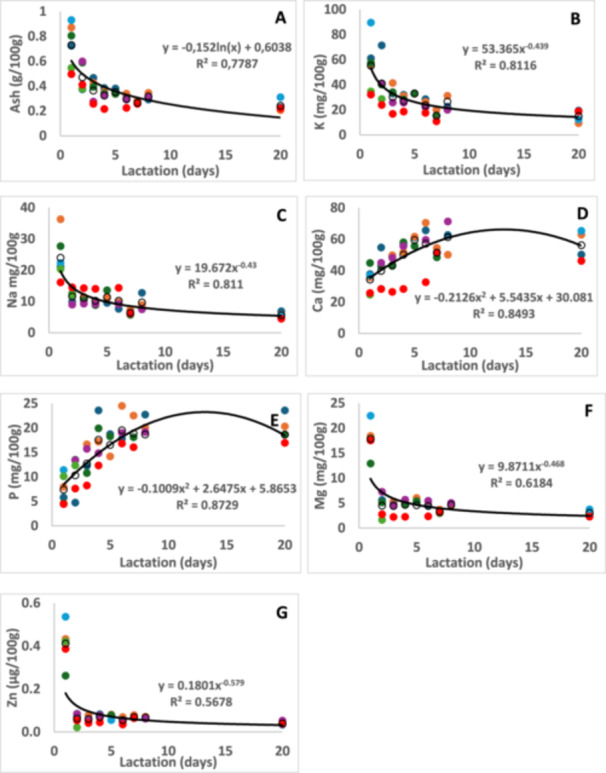
The changes of ash (A), potassium (B), sodium (C), calcium (D), phosphorous (E), manganese (F) and zinc (G) in white rhinoceros milk during early lactation with best‐fit trend lines. The interindividual variation of changes over lactation is shown for each animal: Average (O), Olive (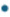), Twinkle (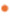), Timbi (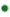), Sibeva (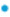), Tana (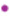), River (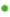), Wyntir (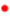).

**Figure 4:**
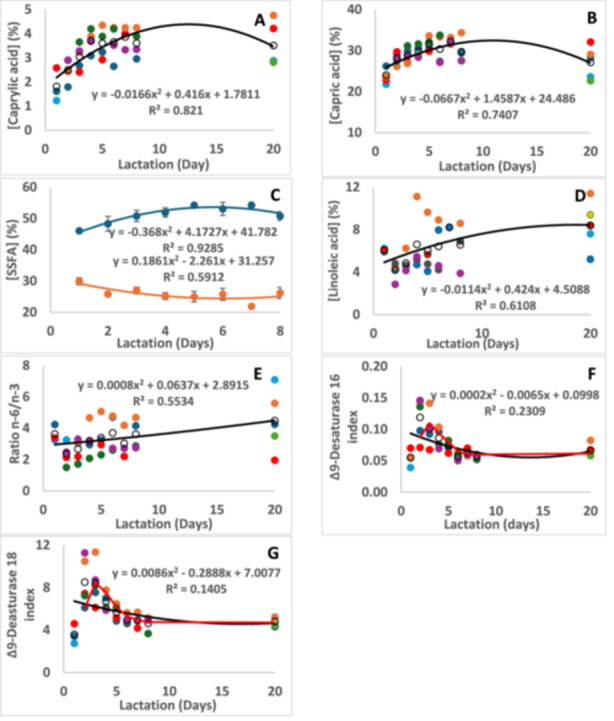
The changes of (A) capric acid, (B) caprylic acid, (C) sum of medium chain fatty acids (SMSFA) (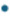) and sum of long chain fatty acids (SLSFA) (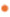), (D) PUFA, (E) ratio n‐6/n3, (F) Δ9‐desaturase 16 index and (G) Δ9‐desaturase 18 index in white rhinoceros milk over lactation with best‐fit trend lines and moving average lines (red) for the desaturase indexes. The interindividual variation of changes over lactation is shown for each animal: Average (O), Olive1 (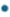), Twinkle (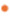), Timbi (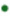), Sibeva (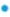), Tana (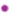), River (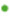), Wyntir (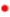).

The average content of the milk components of the white rhinoceroses under study is shown in Table 1. The changes of selected milk components over lactation are depicted in Figures 1, 2, 3, 4. It was not possible to collect milk daily from all seven rhinoceroses due to them hiding their calves in dense bush, hence the difference in number of data points per day. In the figures the trend lines are presented to indicate the statistical significance of the changes while the changes of components for each rhinoceros is shown separately to indicate the interindividual variation. For instance, the change of lactose (Figure 1B) follow a high path in the milk of Olive and a low path in that of Twinkle. The content of ash, K and Ca (Figure 3) in the milk of Wyntir also follow a low path of changes compared to that of the other rhinoceroses.

**Table 1:** The proximate and mineral composition and gross energy (average ± standard deviation) of the milk of six white rhinoceroses at days 1, 2 and 20 of lactation.

Nutrient (g/100 g milk)	Day 1 (Colostrum)	Day 2	Day 20	Indian rhinoceros^a^ (colostrum)
Dry matter	23.8 ± 2.4	11.9 ± 1.3	9.4 ± 1.3	13.8
Ash	0.7 ± 0.2	0.5 ± 0.1	0.2 ± 0.1	0.67
Fat	0.6 ± 0.3	1.7 ± 0.3	1.0 ± 0.3	
Protein	18.0 ± 5.8	3.8 ± 0.7	1.3 ± 0.3	8.53^b^
NPN	0.2 ± 0.1	0.1 ± 0.01	0.1 ± 0.1	
Lactose	5.6 ± 1.5	6.8 ± 0.9	6.6 ± 0.5	3.69
Gross energy (kCal/100 g)	123.2 ± 36.7	61.5 ± 5.2	41.4 ± 6.2	
**Minerals (mg/100 g)**				
Ca	33.4 ± 7.3	38.8 ± 12.0	53.3 ± 15.1	81.0
P	10.3 ± 3.9	14.3 ± 3.5	19.9 ± 2.8	75.0
K	55.2 ± 20.9	41.0 ± 18.5	15.0 ± 4.9	
Mg	14.5 ± 6.2	4.5 ± 2.3	3.0 ± 0.6	
Na	24.0 ± 2.3	11.6 ± 2.2	5.6 ± 1.0	
Cu	0.1 ± 0.0	0.1 ± 0.0	0.1 ± 0,0	
Fe	0.1 ± 0.0	0.1 ± 0.1	0.1 ± 0,0	
Zn	0.4 ± 0.1	0.1 ± 0.0	0.1 ± 0.0	
Mn (µg/100 ml)	0.4 ± 0.1	0.3 ± 0.1	0.3 ± 0,1	

^a^ Gimmel et al. (2018).

^b^ Crude protein content.

The total dry matter (DM) was around 23.8 g/100 g in colostrum (day 1) and decreased to around 11.5 g/100 g on day two and 9.5 g/100 g from day three onward. Some of the components that make up the DM show a slower stabilization of up to 7 days, as will be discussed below. Indeed, the (PCA) of the major components of the rhinoceros colostrum and milk (Figure 2), ash, lactose, fat, protein and (NPN), indicated that colostrum is completely different from the transitional milk. The milk of days 3 to 8 are very similar, also to that of day 20. The milk of day two shows only a partial overlap with the milk of days 3 to 8. The PCA biplot explained 79.3%, of the variation of which dimension 1 explained 59.8% and dimension 2 explained 19.5%. A similar conclusion of changes from colostrum to transitional milk was reached in a metabolomics study of the same white rhinoceros milk samples (Osthoff and Nieuwoudt 2024).

Calculation of GE from the three macronutrients showed that the gross energy of white rhinoceros colostrum was approximately 123 kCal/100 g (Figure 1F; Table 1). It changed to 61.5 kCal/100 g and gradually stabilized between 40 and 50 kCal/100 g milk. A similar trend was reported for the Indian rhinoceros, however, with lower values at 66.9 kCal/100 g colostrum and 34–36 kCal/100 g after day 5 (Gimmel et al. 2018).

The ash content in colostrum was around 0.7 g/100 g, dropped to 0.5/100 g and stabilised between 0.2 and 0.3 g/100 g from day 4. The increase in ash content during lactation is observed in most other species, such as human and mare (Auldist et al. 1998; Shennan and Peaker 2000; Summer et al. 2004). The content of K and Na also showed a reduction from 55.2 to 24.0 g/100 g respectively in colostrum to around 41 and 11.6 g/100 g on day 2 with a further gradual reduction to a half of these amounts on day 7. At day 20 the K and Na content were 15.0 and 5.6 g/100 g, respectively. The change in Mg follows a different profile, from 14.5 g/100 g in cholostrum and stabilizing at around 4 g/100 g from day 2 of lactation. The minerals of minor content follow a similar sharp change, with Zn as example being at 0.4 g/100 g milk stabilizing at 0.1 g/100 g. The increases in K and Na were inversely related to the increase of lactose during the first week of lactation, which is described below, and suggests that the changes in osmotic activity in colostrum is caused by salts, and later by lactose, to control the milk volume (Neville 1999; Shennan and Peaker 2000). The decrease of salts is due to closing of the tight junctions between the epithelial cells that prevents leakage from the blood plasma via the paracellular pathway (Nguyen and Neville 1998). The average Ca content was around 32 mg/100 g colostrum, which gradually increased to stabilise at around 53 mg/100 g transitional milk. This increase is an indication of its colloidal form with the caseins. The P content changed from 10 mg/100 g in colostrum to 20 mg/100 g in transitional milk. The Ca:P ratio is around 2.5:1, which compares with what was found in mature milk (Osthoff et al. 2021).

The lactose is the major component of the white rhinoceros milk and the content increased slightly from 5.6 g/100 g in colostrum to stabilize at an average of between 6 and 7 g/100 g milk from day 4 onward. According to the low R^2^ of the trend line, the change is not significant. Definite changes from colostrum to transitional milk was, however, reached in a metabolomics study of the same white rhinoceros milk samples (Osthoff and Nieuwoudt 2024). A trend of increasing lactose in colostrum to milk from around 3.7% to 10% was, however, observed in the Indian rhinoceros (Gimmel et al. 2018). Similar increases were also reported for other perissodactyls such as horse (Solaroli et al. 1993) and metabolomic studies of donkey milk (Garhwal et al. 2023; Yang et al. 2024).

The protein content of the rhinoceros colostrum was 18.0%, decreased to 3.8% on day two and stabilized around 1.3% from day 3. Such decrease was also reported for the Indian rhinoceros (Gimmel et al. 2018). The NPN content also gradually decreased from around 0.2% in colostrum to stabilize at 0.9% after 6 days. It had been established that the high protein content in colostrum is due to the presence of immunoglobulins and lactoferrin (Lewis‐Jones et al. 1985; Pecka et al. 2011). Their concentrations decrease rapidly after day 2, when the secretion of caseins is fully established and their concentration stabilised.

According to the trend line, the changes in fat content from colostrum to transitional milk is not significant. However, the moving average shows the fat content to peak at days 3 and 4 (Figure 1D and Table 2). This seems to coincide with changes of the fatty acid composition of the milk fat, which is described below.

In the rhinoceroses under study, the capric‐, lauric‐, myristic‐, and palmitic acids are the major fatty acids in the milk fat, followed by the unsaturated oleic‐ and linoleic acids. This differs from our previous report where capric‐, lauric‐, palmitic‐ and linoleic acids were the major ones (Osthoff et al. 2021). The difference may lie in the dietary intake of more fresh grass from the subtropical environment, compared to that of the dry Southern Kalahari environment of the previous study. In the milk of the Indian rhinoceros a change in fatty acid was observed when fresh grass was included in the diet (Gimmel eta l. 2018). In the milk of horse (Hoffman et al. 1998; Markiewicz‐Keszycka et al. 2014) and donkey (Martini et al. 2015) it was found that fresh grass and foliage played the greatest role, because they contained more long chain fatty acids than hay and were responsible for higher amounts thereof in the milk fat. Nutrition may therefore also affect the fatty acid composition of the white rhinoceros milk under study, because parturition of all animals was at different months and seasons. However, no such correlation was observed in the milk samples of the current study. The reason might be the availability of fresh grass throughout the year. Furthermore, although butyric and hexanoic acids were reported in small amounts in milks of the Equidae and lowland tapir, they were absent in the rhinoceroses (Gimmel et al. 2018; Osthoff et al. 2021). The reason might be because the absorbance of volatile products produced by fermenting bacteria in the hindgut of the Rhinocerotidae is not as successful as in the Equidae or that the conversion of butyrate in the liver is faster. Fatty acids longer than 18 carbons were not detected in the white rhinoceros milk under study. It is not clear whether they have not been detected or whether they were unavailable in the food.

The content of caprylic‐ and capric acids gradually increased from 1.8% and 25.9% respectively in colostrum to stabilize at 3.6% and 31.5% at day 5 (Figure 4A,B and Table 3). The sum of the medium chain fatty acids (SMSFA; carbon length 8–12) also increased significantly (Figure 4C) while the sum of the long chain fatty acids (SLSFA; carbon length 12–18) decreased. Such a change had been observed in the Indian rhinoceros (Gimmel at al. 2018) as well as other species such as the cow (Wilms et al. 2022; O'Callaghan et al. 2020), ewe (Povlikova et al. 2010), sow (Csapo et al. 1996; Ren et al. 2022) and human (Kovács et al. 2005; Kuipers et al. 2012), but not any of the Perissodactyla.

**Table 2:** Fat content and Δ9‐Desaturase indexes of white rhinoceros milk on peak days compared to days 7 and 8.

Fat property	Amount*
Fat content days 1 (g/100 g)	0.6 ± 0.3^a^
Fat content days 3 + 4 (g/100 g)	1.6 ± 0.6^b^
Fat content days 7 + 8 (g/100 g)	0.8 ± 0.3^a^
Δ9‐Desaturase 16 index day 1	0.06 ± 0.02^c^
Δ9‐Desaturase 16 index days 2 + 3	0.11 ± 0.03^d^
Δ9‐Desaturase 16 index days 7 + 8	0.06 ± 0.01^c^
Δ9‐Desaturase 18 index day 1	3.58 ± 0.94^c^
Δ9‐Desaturase 18 index days 2 + 3	8.44 ± 1.96^d^
Δ9‐Desaturase 18 index days 7 + 8	4.34 ± 0.68^c^

* Superscripts a and b present significant differences at *p* ≤ 0.005 and c and d at *p* ≤ 0.001.

**Table 3:** Comparison of the fatty acid composition and desaturase activities energy (average ± standard deviation) of the milk of white rhinoceroses at days 1 and 5 of lactation and with rhinoceroses that lived in different nutritional conditions.

FAME (% of total fatty acids)	Abbreviation	Day 1* (Colostrum)	Day 5*	Osthoff et al. (2021)
Butyric	C4:0	N.D.	N.D.	N.D.
Caproic	C6:0	N.D.	N.D.	N.D.
Caprylic	C8:0	1.8 ± 0.6^a^	3.6 ± 0.6^b^	1.8 ± 0.5
Capric	C10:0	25.9 ± 4.0^a^	31.5 ± 1.7^b^	17.2 ± 4.3
Hendecanoic	C11:0	1.5 ± 1.3	0.3 ± 0.3	N.D.
Lauric	C12:0	21.4 ± 1.8	19.1 ± 2.3	10.9 ± 3.0
Tridecoic	C13:0	1.4 ± 1.0	0.3 ± 0.3	
Myristic	C14:0	11.2 ± 1.9	9.3 ± 2.5	5.9 ± 1.6
Myristoleic	C14:1c9	0.9 ± 0.6	0.1 ± 0.2	N.D.
Pentadecylic	C15:0	0.2 ± 0.3	0.3 ± 0.2	7.6 ± 2.8
Palmitic	C16:0	14.9 ± 1.3	13.1 ± 1.2	20.5 ± 3.0
Palmitoleic	C16:1c9	0.8 ± 0.2	1.0 ± 0.1	1.1 ± 0.7
Margaric	C17:0	0.1 ± 0.2	0.4 ± 0.1	4.6 ± 1.7
Heptadecenoic	C17:1c10	0.3 ± 0.3	0.2 ± 0.3	0.3 ± 0.1
Stearic acid	C18:0	2.5 ± 1.0	2.0 ± 0.3	5.2 ± 0.9
Oleic	C18:1c9	8.9 ± 1.6	9.9 ± 1.4	10.7 ± 3.9
Vaccenic	C18:1c7	1.00 ± 0.2	1.2 ± 0.3	1.2 ± 0.5
Linolelaidic	C18:2t9,12 (n‐6)	N.D.	N.D.	2.6 ± 1.0
Linoleic	C18:2c9,12 (n‐6)	5.5 ± 1.1	6.0 ± 2.5	5.4 ± 1.7
α‐Linolenic	C18:3c9,12,15 (n‐3)	1.8 ± 0.3	1.8 ± 0.3	4.9 ± 2.3
γ‐Linolenic	C18:3c6,9,12 (n‐6)	N.D.	N.D.	N.D.
Eicosadienoic	C20:2c11,14 (n‐6)	N.D.	N.D.	0.1 ± 0.1
Eicosatrienoic	C20:3c11,14,17 (n‐3)	N.D.	N.D.	0.2 ± 0.1
Arachidonic	C20:4c5,8,11,14 (n‐6)	N.D.	N.D.	0.1 ± 0.1
**Fatty acid ratios**				
Saturated fatty acids		80.9 ± 1.4	79.8 ± 3.3	73.72 ± 5.9
Mono‐unsaturated fatty acids		11.9 ± 1.6	12.4 ± 1.8	13.2 ± 5.1
Poly‐unsaturated fatty acids		7.2 ± 0.9	7.8 ± 2.6	13.1 ± 3.1
Omega‐6 fatty acids		1.8 ± 0.3	1.8 ± 0.3	8.0 ± 1.6
Omega‐3 fatty acids		5.5 ± 1.1	6.0 ± 2.5	5.1 ± 2.4
PUFA/SFA		0.1 ± 0.0	0.1 ± 0.1	
SMCSFA (C8:0 – C12:0)		46.0 ± 1.0^a^	54.2 ± 1.3^b^	
SLCSFA (C14:0 – C18:0)		30.6 ± 2.3^a^	25.0 ± 3.4^b^	
n‐6/n‐3		3.2 ± 1.1	3.4 ± 1.2	
Δ9‐Desaturase 14 Index		0.08 ± 0.06	0.01 ± 0.02	0
Δ9‐Desaturase 16 Index		0.06 ± 0.02	0.07 ± 0.01	0.05 ± 0.04
Δ9‐Desaturase 18 Index		4.53 ± 2.54	5.64 ± 0.76	2.23 ± 0.69

* Superscripts a and b indicate significant differences at *p* ≤ 0.001 between days 1 and 5.

Regarding the unsaturated fatty acids, an increase in linoleic acid content was noted according to the R^2^ of 0.6108, however, the change did not occur in all animals (Figure 4D). An increase in linoleic acid within the first days of lactation was also observed in two Indian rhinoceroses (Gimmel et al. 2018), while no changes or increases were reported in other species such as the cow (Wilms et al. 2022; O'Callaghan et al. 2020), ewe (Pavlíková et al. 2010), sow (Ren et al. 2022) and human (Kovács et al. 2005; Kuipers et al. 2012).

A more detailed interpretation of the monounsaturated fatty acids regarding their desaturation by the mammary Δ9 desaturase activity to form myristic‐, palmitic‐ and stearic acid is revealing. The Δ9‐desaturase index is indicated by the ratio of a Δ9‐unsaturated fatty acid to its saturated equivalent (Figure 4F,G). The Δ9‐desaturase 14 and ‐16 indexes are low and have also been reported as low in our previous study (Osthoff et al. 2021) as well as Indian rhinoceros (Gimmel et al. 2018), horse and donkey (Salimei and Fantuz 2012), plain's zebra (Uniacke‐Lowe and Fox 2011) and Tapir (Van Nieuvenhove et al. 2014). According to the R^2^ of the graphs, no significant changes occurred from colostrum to transitional milk. However, similar as the fat content, peaks were noted at days 2 and 3. The Δ9‐desaturase 16 indexed peaked at 0.11 and the Δ9‐desaturase 18 index at 8.44, which is a two‐fold increase of both from colostrum, with a decrease thereafter. Such changes have not been reported for other species. Nevertheless, the simultaneous changes in fat content, SMSFA's and Δ9‐desaturase indexes indicated that it may take up to 5 days for the fat metabolism of the milk gland cells to stabilize.

It may be concluded that the colostrum phase of the milk secretion in the white rhinoceros ends at day 2 after parturition. During this stage the salt and protein content decreases sharply due to the closure of the tight junctions between the mammary gland epithelial cells, which prevents seepage from the blood (Nguyen and Neville 1998). Simultaneously the transition to milk production starts at this stage as indicated by the gradual increase in Ca, bound to caseins, and lactose, which reach a maximum at day 4. The activation of the fat metabolism is also initiated as shown by the de novo fatty acid synthesis and desaturation of saturated fatty acids, which reaches a maximum at day 5.

Immediate application of our data would be the rearing of rhinoceros calves orphaned by poaching. In South Africa alone, the poaching toll has soared from 36 per year between 1990 and 2007 to 1215 in 2014. This has since decreased to 594 in 2019 and 307 during 2023 (www.savetherhino.org). A formula milk powder for horses (Salvana Fohlenmilch, Salvana Tiernahrung GmbH, Elmsdorf, Germany) combined with whey powder to obtain a fat:protein:lactose ratio of approximately 1:2:4 was used to successfully raise an orphaned white rhinoceros calf (Ludwig et al. 2010). Foal formula milk is currently also used in‐house at Care for Wild Rhino Sanctuary to successfully raise white rhinoceros calves for up to 2 years. Although the natural tolerance of young animals allows for some variation in nutrient composition of milk and surrogate milk, the current results suggest that the medium chain fatty acid content of formula milk should be increased, for which coconut or palm kernel oil might be a source of capric‐, (6%), lauric‐ (48%) and myristic acid (17%) (Scrimgeour, 2005).

## Data Availability

The data that support the findings of this study are available from the corresponding author upon reasonable request.
